# Characterizing Precision Nutrition Discourse on Twitter: Quantitative Content Analysis

**DOI:** 10.2196/43701

**Published:** 2023-10-12

**Authors:** Sapna Batheja, Emma M Schopp, Samantha Pappas, Siri Ravuri, Susan Persky

**Affiliations:** 1 Department of Nutrition and Food Studies George Mason University Fairfax, VA United States; 2 Social and Behavioral Research Branch National Human Genome Research Institute Bethesda, MD United States

**Keywords:** nutrigenetics, nutrigenomics, precision nutrition, Twitter, credibility, misinformation, content analysis

## Abstract

**Background:**

It is possible that tailoring dietary approaches to an individual’s genomic profile could provide optimal dietary inputs for biological functioning and support adherence to dietary management protocols. The science required for such nutrigenetic and nutrigenomic profiling is not yet considered ready for broad application by the scientific and medical communities; however, many personalized nutrition products are available in the marketplace, creating the potential for hype and misleading information on social media. Twitter provides a unique big data source that provides real-time information. Therefore, it has the potential to disseminate evidence-based health information, as well as misinformation.

**Objective:**

We sought to characterize the landscape of precision nutrition content on Twitter, with a specific focus on nutrigenetics and nutrigenomics. We focused on tweet authors, types of content, and presence of misinformation.

**Methods:**

Twitter Archiver was used to capture tweets from September 1, 2020, to December 1, 2020, using keywords related to nutrition and genetics. A random sample of tweets was coded using quantitative content analysis by 4 trained coders. Codebook-driven, quantified information about tweet authors, content details, information quality, and engagement metrics were compiled and analyzed.

**Results:**

The most common categories of tweets were precision nutrition products and nutrigenomic concepts. About a quarter (132/504, 26.2%) of tweet authors presented themselves as science experts, medicine experts, or both. Nutrigenetics concepts most frequently came from authors with science and medicine expertise, and tweets about the influence of genes on weight were more likely to come from authors with neither type of expertise. A total of 14.9% (75/504) of the tweets were noted to contain untrue information; these were most likely to occur in the nutrigenomics concepts topic category.

**Conclusions:**

By evaluating social media discourse on precision nutrition on Twitter, we made several observations about the content available in the information environment through which individuals can learn about related concepts and products. Tweet content was consistent with the indicators of medical hype, and the inclusion of potentially misleading and untrue information was common. We identified a contingent of users with scientific and medical expertise who were active in discussing nutrigenomics concepts and products and who may be encouraged to share credible expert advice on precision nutrition and tackle false information as this technology develops.

## Introduction

### Background

Precision nutrition has attracted a great deal of attention and investment as a promising direction for scientific and health care development from many corners of industry, government, academia, medicine, media, and beyond [1-4]. This research focuses on the interplay between genetics, personal biology, microbiome, social and environmental exposures, and dietary behavior and how this collection of factors relates to health outcomes. Much of the excitement underlying precision nutrition rests on the long-understood fact that responses to dietary behaviors are variable and can depend on individual differences rooted in both genetic makeup and how genes interact with a broader environment [5]. It is predicted that tailoring dietary approaches to an individual’s genomic profile could provide optimal dietary inputs for biological functioning and support adherence to dietary management protocols [6]. The anticipated benefits of implementing precision nutrition approaches range from general improvement in dietary quality to enhanced weight loss and long-term weight management, to improvements in specific biomarker profiles such as cholesterol and HbA_1c_, to dietary treatment of diseases such as diabetes and cardiovascular disease.

**Figure 1:**
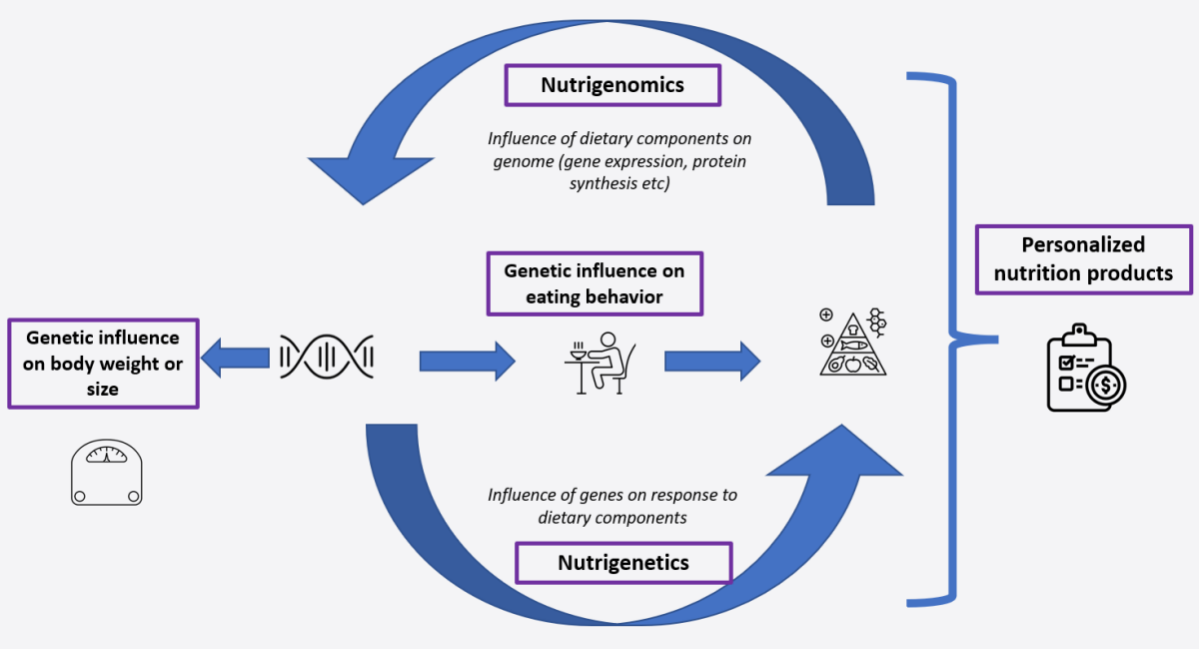
Framework depicting concepts included in this study.

Under the broader umbrella of precision nutrition, the use of an individual’s genetic makeup to make personalized dietary recommendations has long been known as *nutrigenetics* [6]. A related concept, *nutrigenomics*, is broadly understood as the study of gene-diet interactions but is most often used to refer more specifically to the effects of nutrients on the genome, such as on gene expression [6]. Therefore, in one sense, nutrigenetics can be subsumed under the broader term nutrigenomics, but in practice, most often the terms are discussed as complementary areas of study that both comprise precision nutrition (Figure 1). We used these terms in the latter way in this study.

Given the potential promise that genomics-based precision nutrition may improve difficult problems of long-term dietary management, researchers, practitioners, and industry entities have worked toward the development of evidence-based approaches. Although progress has been made in this regard, trials of nutrigenetics-oriented dietary approaches have been mixed with regard to their efficacy [7-11]. Similarly, there is little evidence to support claims made regarding the potential benefits of nutrigenomics. Few studies have investigated the role of dietary changes or supplements on epigenetic processes, and existing studies have often been of lower quality [12].

Overall, reviews of existing evidence and professional organization consensus statements suggest that the evidence for the inclusion of genetic data in dietary tailoring is weak and insufficient at present, and that far more evidence is needed before DNA-based dietary tailoring could be recommended for health or medical practice [12-15]. Research has also suggested that the translation pipeline to bring nutrigenetics into health care will require a great deal of education and resources before clinical use is feasible [16]. Globally, most experts assert that the translation of nutrigenetics and nutrigenomics to patients and consumers is premature [17-19].

Despite their scientific prematurity, the application of precision nutrition approaches is appealing to stakeholders because many current dietary approaches for weight control and disease management lack efficacy over the long term [20]. As such, the promise of precision nutrition has received media and public attention [21-23]. Public interest in engaging in precision nutrition approaches has been reported to be relatively high [8,24], reflecting largely positive attitudes about this technology that tend to eclipse concerns [25,26]. The nature of this attention has led researchers to deem precision nutrition as a source of “hype”; an area where expectations for technology far exceed reality [27]. This is perhaps unsurprising given the considerable and continuing hype that has been ascribed to the use of many genetic technologies in health and medicine [28]. Such premature translation often occurs under the umbrella of direct-to-consumer genetic testing, wherein health-relevant genetic information in areas from disease risk to athletic ability has been provided to consumers, sometimes in the absence of a solid evidence base [29]. As such, nutrigenetics and nutrigenomics can be considered as another piece of the larger tendency for genomics technologies to be prematurely translated in consumer settings.

The nutritional arena is also rife with hype. Many individuals seek dietary approaches and supplements as cures for chronic diseases, some promising miracle results or magic bullets [30]. Such supplements are typically also offered directly to consumers and often have high financial costs; claims made regarding these products rarely address safety [31]. The combination of genomics, dietary supplements, and the weight loss industry could create a perfect storm for the rapid growth of precision nutrition products, regardless of efficacy. Since the early 2000s, several companies have promised improved health and wellness outcomes through tailored diets and micronutrient supplementation based on direct-to-consumer testing [19]. Most of these offerings are currently based on unregulated tests and have not been rigorously evaluated [32,33].

The footprint of precision nutrition promotion, information, and discussion on social media has not yet been investigated. Product website investigations have found insufficient information or transparency about precision nutrition products, such that consumers are unlikely to be able to make informed purchasing decisions [34-36]. Although product websites are a frequent source of information, consumers also seek out discussions about health products on expert sites, mass media, and social media. Social media is perceived to provide first-hand testimonials, alternative options, more in-depth information, and social support [37]. Social media is also a frequent source of health information because its use is already integrated into people’s daily lives and routines [37,38]. When individuals seek independent information on these topics, it is unclear what information they will encounter, how discussions are framed, who is involved in these conversations, and how accurate the information is likely to be. Looking at the related topic of precision medicine, research has shown information presence across media platforms with discourse commonly occurring on social media [39]. Similarly, discourse on nutrition, weight loss, fitness, and related topics is frequently found on social media [40]. Thus, when evaluating discourse related to precision nutrition, social media is a sensible place to begin.

Among the popular social media platforms, Twitter is promising for health surveillance and research for a number of reasons. It is one of the most frequently studied social media platforms in health research and practice [41]. It is also an active venue for users from a wide variety of contexts, including scientists and medical professionals [42,43]. Twitter’s sharing of outside resources contributes to conversations and supports active discourse in response to resources and information. It is largely an open network, which increases the sharing of content among experts, individuals, and communities [44,45]. Twitter is a readily available tool for increasing education and awareness of evidence-based information; it has hosted many health-oriented campaigns in the past [41,46,47]. A systematic examination of publicly available precision nutrition information on Twitter can illuminate areas that might benefit from campaigns to educate consumers or misconceptions that need to be addressed. Indeed, Twitter and other social media platforms are known for the spread of misinformation, dubious marketing, and overblown claims [48], all of which are concerns in the precision nutrition domain. Information about naturally arising areas of interest in social media communication related to precision nutrition can also guide communication approaches if and when precision nutrition is ready for evidence-based implementation.

### Objective

This study aimed to characterize the landscape of precision nutrition in the form of nutrigenetics and nutrigenomics discourse on Twitter through several broad research questions:

1. What is the balance between the various topics that arise when nutrigenetics- and nutrigenomics-related concepts are discussed? Do information characteristics such as credibility, benefit framing, citation of sources, association with products, and community engagement metrics vary by topic?
2. Who is involved in these discussions—what types of expertise are evident among the communicators? Are these expertise types associated with variations in the information characteristics and topics?
3. How often do communications contain potentially misleading or false information? Do potential misleading or false communications co-occur with other message characteristics? What types of communication tend to contain potentially misleading or false information?

## Methods

### Codebook

The study team created a codebook for quantitative content analysis based on the research questions and the open coding of a random sample of tweets. The codebook was refined during the coder training process. The codebook contains several items, including eligibility for the data set, tweet topic, information about the tweet’s author, content details, and engagement metrics. To be eligible for coding, a given tweet was required to be written in English and to contain the concepts of human genetics and food, eating, or body weight or size. Any quoted tweets were considered part of the tweet unit and were considered in light of the focal tweet. Linked material (eg, an outside website) that was not visible as part of the tweet body was not included in the tweet unit, although it was considered for coding items related to the existence of product links and informational resources. Information from the tweet authors’ bio was not fact-checked and was coded as written. More details on the codebook can be found in Table 1, and the full codebook is provided in Table S1 in Multimedia Appendix 1.

**Table 1:** Coding categories and definitions.

Item			Options		Definition
Topic			Precision nutrition product Nutrigenetics concepts Nutrigenomics concepts Genes influence eating behavior Genes influence body weight or size Other		Main theme of the tweet content; categories are mutually exclusive
**Author-relevant**					
	Author expertise	Science and medical Wellness Neither		On the basis of author-provided biographical information; mutually exclusive such that authors with both science and medical and wellness were coded in science and medical. Although wellness expertise is less well-defined, this category included authors who stated or insinuated special knowledge about wellness or one of its subcategories (eg, diet and supplements). Example occupational categories categorized as wellness expertise include naturopath, personal trainer, and health coach.	
	Referenced professional credentials	Yes or no		On the basis of author-provided biographical information; relevant medical or scientific credentials (eg, MD, PhD)	
	Author affiliation	Business Academic or nonprofit Individual (unaffiliated) Media outlet Other		On the basis of author-provided biographical information; mutually exclusive. Used bio-provided links where applicable	
**Content-relevant**					
	References advantages of personalized nutrition	Yes or no		States something specific and positive about personalized nutrition products and concept applications	
	References problems of personalized nutrition	Yes or no		States something specific and negative or detrimental about personalized nutrition products and concept applications	
	Product link	Yes or no		Contains a direct link to a personalized nutrition product	
	Information source link	Yes or no		Contains a direct link to a secondary information source	
	Misleading or incorrect information	Potentially misleading Incorrect information Neither		Determined by secondary coding process and fact-checking where needed	
	Commercial entity identity	Name of commercial entity referenced in tweet		Names of companies or businesses mentioned in the tweet or linked to were recorded and tabulated	
**Engagement**					
	Replies	None <10 ≥10		Number of direct replies to tweet; binned into 3 groups (active tweets only)	
	Retweets	Raw number of retweets; including quote tweets		Number of retweets present on tweet at time of data capture (active tweets only)	
	Likes	Raw number of likes		Number of likes present on tweet at time of data capture (active tweets only)	
	Followers	Raw number of followers for tweet author		Number of followers present in author bio at the time of data capture (active tweets only)	

A total of 4 trained coders assessed all tweets for each item in the codebook, with the exception of potentially misleading and incorrect information. All coding variables at this stage achieved a benchmark of Krippendorff α of .67 or higher on 10% of the data set. The remaining tweets were assessed for these variables using a single trained coder. When tweets were still available on the Twitter platform, they were accessed on the web and used directly for coding. When tweets were deleted from Twitter before the coding process, information from Twitter Archiver was used to execute the coding process. Some categories (eg, engagement metrics) cannot be coded when tweets are no longer present on the platform. Engagement metrics were captured at the time of coding, ensuring that tweets were at least 2 weeks old and thus unlikely to receive further engagement.

Following the initial coding, SB and SP, experts on genetics and nutrition, assessed all tweets for the presence of potentially misleading information, and further denoted whether the tweet contained false information. When this was unclear, the tweets were fact-checked by SB and SP. All disagreements were resolved through discussion considering scientific literature (or other applicable sources where nonscientific statements were addressed). The type of misleading or false information was also assessed according to a set of 8 categories generated following the initial coding process (Multimedia Appendix 1). All tweets were double-coded for these variables, and in all cases, the κ values for intercoder agreement reached or exceeded the benchmark value of 0.6.

### Data Source

We used Twitter Archiver [49] to capture all tweets over a 3-month period from September 1, 2020, to December 1, 2020, which contained combinations of keywords represented by the search string ([genes OR genetics OR DNA OR nutrigenomics OR nutrigenetics] AND [diet OR nutrition OR eating OR food OR slimming]) to capture tweets related to the intersection of genetics and diet. This process resulted in 6366 tweets. A random sample of 1200 tweets was created by sorting the data according to a random number. Selected tweets were evaluated to determine whether they pertained to the relationship between food and genes; those that did not meet this criterion were excluded from further coding. This process was executed by trained coders as part of the coding process and was subject to intercode agreement benchmarks. Of the 1200 tweets, the final coding data set contained 504 (42% random sample) tweets.

### Ethical Considerations

This study was deemed exempt by the relevant offices at the National Human Genome Research Institute (000388) and George Mason University (1726834-1). To support Twitter users’ anonymity, the current report does not contain any identifying information or direct quotes in accordance with published guidelines and recommendations [50].

### Data Analysis

All descriptive statistics were generated and some coding categories containing few tweets were merged or omitted from the analysis. Differences between groups were assessed using omnibus chi-square analysis with Bonferroni-corrected planned contrasts. Engagement metrics coded on a continuous scale exhibited significant skew and were assessed using Kruskal-Wallis tests with Bonferroni-corrected pairwise comparisons.

## Results

### Results by Tweet Category

**Figure 2:**
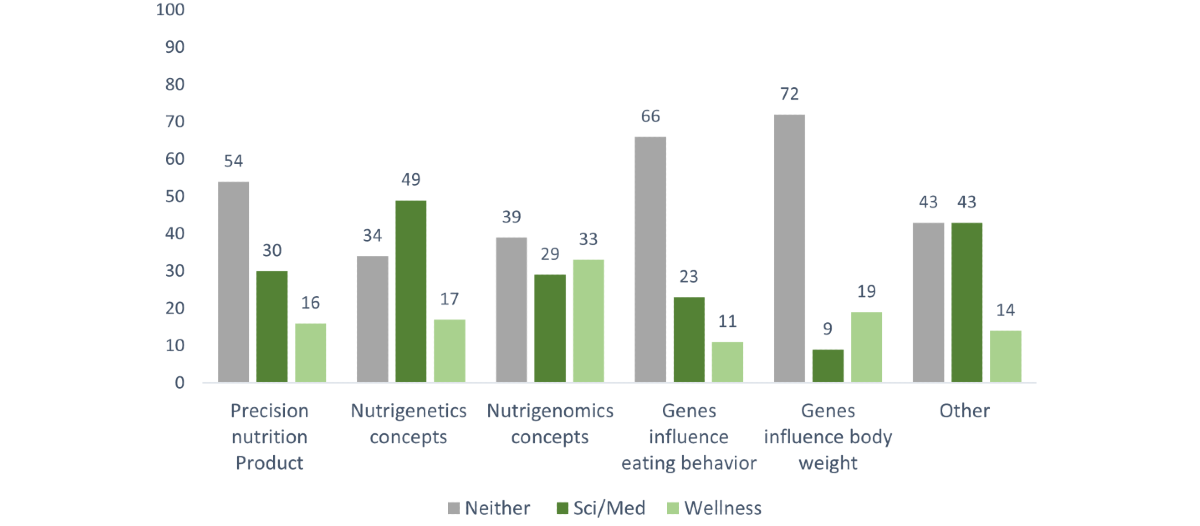
Percentage of tweet authors claiming each expertise type by content type.

Full results are presented in Table 2. Key findings included that tweets about a precision nutrition product and those about nutrigenomics were the most common categories, followed by those about genes influencing body weight, genes influencing eating behavior, and nutrigenetics concepts (see Table 2 for all frequencies). Author expertise differed significantly by category, wherein tweets regarding nutrigenetics concepts were more likely to come from authors with science and medicine expertise (Figure 2). Tweets about nutrigenomics and the influence of genes on body weight were especially unlikely to come from those with science and medicine expertise. Tweets related to products were especially likely to discuss advantages of nutrigenetics or omics and were also more likely to discuss disadvantages or problems. They were also more likely to be linked to a product. In terms of engagement, tweets about genetic influences on weight received more replies and likes than some other categories.

**Table 2:** Characteristics by topic category. Superscript symbols denote values that are significantly different from one another within each variable type.

			Precision nutrition product	Nutrigenetics concepts	Nutrigenomics concepts	Genes influence eating behavior	Genes influence body weight or size	Other	Total, n	Omnibus chi-square or Kruskal-Wallis test (*df*)		*P* value	
Total, n (%)			128 (25)	47 (9)	130 (26)	73 (14)	112 (22)	14 (3)	504	—^a^		—	
**Author-relevant, n (%)**													
	**Author expertise**										57.4 (10)		<.001
		Neither	69 (54)^†,‡,§,∆^	16 (34)^§,∆^	50 (39)^‡,∆^	48 (66)^†,Ω^	81 (72)^Ω^	6 (43)^†,‡,§,∆,Ω^	268				
		Sci or Med	38 (30)^†^	23 (49)^†^	38 (29)^†^	17 (23)^†,‡^	10 (9)^‡^	6 (43)^†^	132				
		Wellness	21 (16)^†^	8 (17)^†,‡^	42 (33)^‡^	8 (11)^†^	21 (19)^†,‡^	2 (14)^†,‡^	99				
	Referenced professional credentials		21 (16)^‡^	26 (55)^‡^	63 (49)^‡^	32 (44)^‡^	8 (7)^‡^	6 (43)^†^	156	81.3 (5)		<.001	
**Content-relevant, n (%)**													
	References advantages of personalized nutrition		50 (39)^‡^	11 (23)^†^	38 (29)^†^	9 (12)^‡^	7 (6)^‡^	1 (7)^†^	116	45.9 (5)		<.001	
	References problems of personalized nutrition		12 (9)^‡^	2 (4)^†^	2 (2)^†^	1 (1)^†^	2 (2)^†^	0 (0)^†^	19	15.8 (5)		.007	
	Product link		42 (33)^‡^	10 (21)^†^	16 (12)^†^	0 (0)^‡^	2 (2)^‡^	4 (29)^†^	74	65.1 (5)		<.001	
	Information source link		39 (31)^‡^	29 (62)^‡^	71 (55)^‡^	37 (51)^‡^	15 (14)^‡^	8 (57)^†^	199	63.5 (5)		<.001	
**Engagement**													
	**Replies, n (%)**										30.3 (10)		.007
		None	109 (90)^†^	36 (88)^†,‡^	102 (86)^†^	56 (89)^‡^	56 (66)^‡^	11 (92)^†,‡^	370				
		<10	9 (8)^†^	5 (12)^†,‡^	15 (13)^†,‡^	5 (10)^‡^	24 (28)^‡^	1 (8)^†,‡^	60				
		≥10	2 (2)^†^	0 (0)^†^	1 (1)^†^	1 (2)^†^	4 (5)^†^	0 (0)^†^	9				
	Retweets, mean (SD)		1.04 (5.35)^†^	0.46 (0.98)^†^	1.01 (3.34)^†^	0.52 (1.12)^†^	1.53 (5.24)^†^	2.50 (3.97)^†^	1.04	5.2 (5)		.39	
	Likes, mean (SD)		3 (13.5)^‡^	2.8 (6.08)^†,§^	3.37 (12.04)^†,‡,§^	2.51 (7.60)^†,‡^	8.34 (19.6)^§^	6.17 (9.98)^†,‡,§^	4.13	28.2 (5)		<.001	
	Followers, mean (SD)		30,091.6 (213,075.8)^†^	1491.7 (2170.5)^†,‡^	7737.00 (39,192.48)^‡^	4642.86 (14,033.07)^†,‡^	4532.07 (20,851.92)^‡^	1864.93 (2386.91)^†,‡^	11,449.58	14.9 (5)		.010	

^a^Not available.

### Results by Expertise Type

Most tweet authors presented themselves as neither science and medicine nor wellness experts, followed by those who were science and medicine experts, and then wellness experts (Table 3). Differences by expertise type were also found for the authors’ stated affiliations, whether the authors referenced any professional credentials and whether there was a link to an information source. Analyses also revealed that authors with neither expertise type were especially unlikely to mention the advantages of nutrigenetics or omics and tended to post fewer outside links. There were no differences by expertise type in terms of mentioning the disadvantages. In terms of engagement, authors with science or medical expertise had significantly more retweets and likes than authors without either type of expertise. There was no difference between the groups in terms of follower counts or replies.

**Table 3:** Characteristics by author expertise. Superscript symbols denote values that are significantly different from one another within each variable type.

				Neither		Science and Medicine		Wellness		Total		Omnibus χ^2^ or Kruskal-Wallis test (*df*)				*P* value		
Total, n (%)				270 (54)		132 (26)		102 (20)		504		—^a^				—		
**Topic category, n (%)**														57.4 (10)				<.001
	Precision nutrition product		69 (26)^†^		38 (29)^†^		21 (21)^†^		128									
	Nutrigenetics concepts		16 (6)^†^		23 (17)^‡^		8 (8)^†,‡^		47									
	Nutrigenomics concepts		50 (19)^†^		38 (29)^†,‡^		42 (41)^‡^		128									
	Genes influence eating behavior		48 (18)^†^		17 (13)^†^		8 (8)^†^		73									
	Genes influence body weight or size		81 (30)^†^		10 (8)^‡^		21 (21)^†^		112									
	Other		6 (2)^†^		6 (5)^†^		2 (2)^†^		13									
**Author-relevant, n (%)**																		
	**Affiliation**												106.6 (6)				<.001	
		Business	16 (6)^†^		36 (27)^‡^		31 (30)^‡^		83									
		Academic or nonprofit	6 (2)^†^		23 (17)^‡^		2 (2)^†^		30									
		Individual (unaffiliated)	230 (86)^†^		57 (43)^‡^		65 (64)^§^		352									
		Media outlet	15 (6)^†^		16 (12)^†^		4 (4)^†^		35									
	Referenced professional credential		49 (18)^‡^		75 (57)^‡^		32 (31)^†^		156		61.7 (2)				<.001			
**Content-relevant, n (%)**																		
	References advantages of personalized nutrition		38 (14)^†^		46 (35)^‡^		32 (31)^‡^		116		26.6 (2)				<.001			
	References problems of personalized nutrition		12 (4)^†^		5 (4)^†^		2 (2)^†^		19		1.3 (2)				.53			
	Product link		18 (7)^†^		29 (22)^‡^		27 (26)^‡^		74		30.6 (2)				<.001			
	Information source link		63 (23)^†^		82 (62)^‡^		54 (53)^‡^		199		65.1 (2)				<.001			
**Engagement**																		
	**Replies, n (%)**												9.1 (4)				.59	
		None	171 (80)^†^		117 (92)^‡^		82 (84)^†,‡^		370									
		<10	37 (17)^†^		9 (7)^‡^		14 (14)^†,‡^		60									
		≥10	6 (3)^†^		1 (1)^†^		2 (2)^†^		9									
	Retweets, mean (SD)		0.45 (1.59)^†^		2.39 (7.06)^‡^		0.57 (1.64)^‡^		1.04		28.8 (2)				<.001			
	Likes, mean (SD)		3.58 (11.09)^†^		6.61 (19.89)^‡^		2.11 (4.63)^†,‡^		4.13		9 (2)				.01			
	Followers, mean (SD)		3148.65 (13,359.80)^†^		33,865.11 (211,231.49)^†^		4106.40 (9669.85)^†^		11,439.58		0.4 (2)				.81			

^a^Not available.

### Results by Presence of Potentially Misleading or Untrue Content

Overall, 29% (146/504) of tweets were judged to contain potentially misleading information, 14.9% (75/504) contained untrue information, and the remaining 56.2% (283/504) had neither (Table 4). The presence of potentially misleading and untrue content both varied significantly by topic category such that posts about products were especially likely to be potentially misleading. Posts about nutrigenomics were especially likely to contain untrue information. The presence of misleading or untrue information was significantly different according to the author’s expertise and affiliation. Those with science and medical expertise had fewer tweets with untrue information than those with wellness expertise; there was no difference between the two in rates of potentially misleading information. Posts discussing the advantages of personalized medicine were more likely to contain potentially misleading or untrue information. There were few differences in the rates of engagement; the only differences emerged in the number of retweets. Tweets without misleading or incorrect information averaged more retweets.

**Table 4:** Characteristics by potentially misleading and untrue content. Superscript symbols denote values that are significantly different from one another within each variable type.

					Potentially misleading			Untrue			Neither			Omnibus chi-square (*df*)				*P* value	
Total, n (%)					146 (29)			75 (15)			283 (56)			—^a^				—	
**Topic category, n (%)**															82.85 (10)				<.001
	Precision nutrition product			50 (34)^†^			19 (25)^†,‡^			59 (21)^†,‡^									
	Nutrigenetics concepts			10 (7)^†,‡^			7 (9)^†,‡^			30 (11)^‡,§^									
	Nutrigenomics concepts			57 (39)^‡^			32 (43)^†,‡^			41 (15)^†^									
	Genes influence eating behavior			7 (5)^‡^			3 (4)^†^			63 (22)^§^									
	Genes influence body weight or size			21 (14)^‡^			14 (19)^†,‡^			77 (27)^§^									
	Other			1 (1)^‡^			0 (0)^†,‡^			13 (5)^§^									
**Author-relevant, n (%)**																			
	**Author expertise**														10.90 (6)				.03
		Neither	75 (51)^†^			37 (49)^†,‡^			158 (56)^†^										
		Science and medical	38 (26)^†^			14 (19)^‡^			80 (28)^†^										
		Wellness	33 (23)^†^			24 (32)^†^			45 (16)^‡^										
	**Afflation**														14.94 (6)				.02
		Business	30 (21)^†^			17 (23)^†^			36 (13)^†^										
		Academic or nonprofit	6 (4)^†^			0 (0)^‡^			25 (9)^‡^										
		Individual (unaffiliated)	98 (68)^†^			52 (70)^†,‡^			202 (72)^†,‡^										
		Media outlet	11 (8)^†^			5 (7)^†,‡^			19 (7)^†,‡^										
	Referenced professional credentials			45 (31)^†^			18 (24)^†^			93 (33)^‡^			2.176 (2)				.34		
**Content-relevant, n (%)**																			
	References advantages of personalized nutrition			59 (40)^†^			25 (33)^†^			32 (11)^†^			51.34 (2)				<.001		
	References problems of personalized nutrition			6 (4)^†^			1 (1)^†^			12 (4)^†^			1.45(2)				.49		
	Product link			36 (25)^†^			17 (23)^†^			21 (7)^†^			27.16 (2)				<.001		
	Information source link			60 (41)^†^			26 (35)^†^			113 (40)^†^			0.97 (2)				.62		
**Engagement**																			
	**Replies, n (%)**														2.08 (4)				.72
		None	109 (83)^†^			58 (81)^†^			203 (84)^†^										
		<10	18 (14)^†^			9 (13)^†^			33 (14)^†^										
		≥10	4 (3)^†^			0 (0)^†^			5 (2)^†^										
	Retweets, mean (SD)			0.70 (2.71)^†^			0.55 (1.57)^†^			1.36 (5.10)^†^			6.93 (2)				.03		
	Likes, mean (SD)			3.92 (13.35)^†^			2.37 (5.94)^†^			4.73 (14.93)^†^			2.74 (2)				.25		
	Followers, mean (SD)			21,163.83 (176,874.04)^†^			3560.33 (9747.47)^†^			8537.85 (72,623.91)^†^			5.34 (2)				.07		

^a^Not available.

### Results for Type of Potentially Misleading or Untrue Content

**Figure 3:**
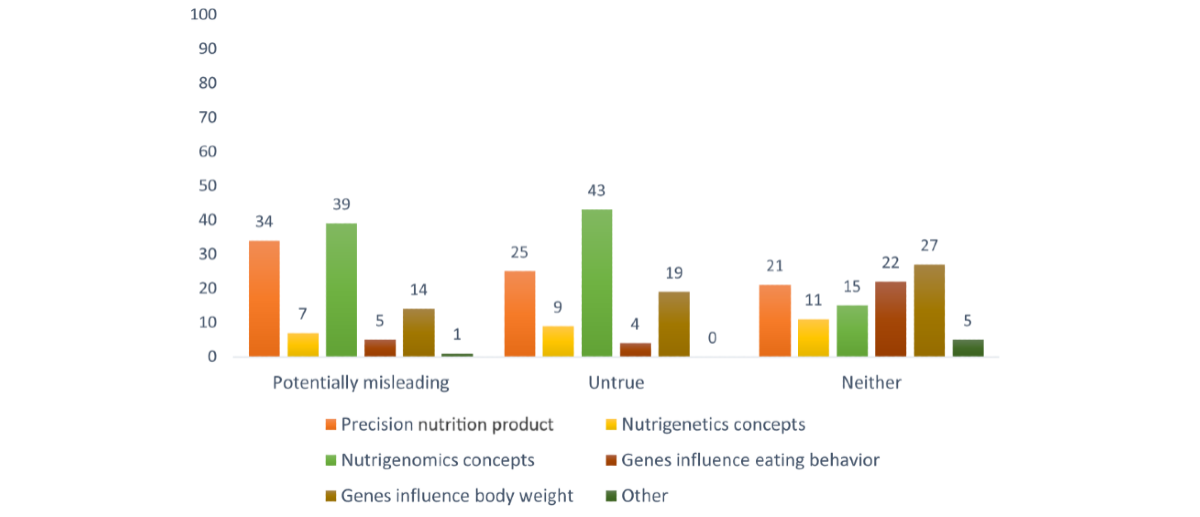
Percentage of tweets from each content type by presence of potentially misleading or untrue content.

Potentially misleading content most frequently related to assertions that personalized nutrition approaches were ready for application to individuals for dietary tailoring. Other frequent topics related to the notion that diet or food can cause changes in one’s genes and that one’s body weight is generally influenced by a single, overriding factor (Figure 3). Tweets containing untrue content were mostly related to the assertion that specific diets or foods can be used to improve DNA (Table 5).

**Table 5:** Categories of potentially misleading or untrue content. Superscript symbols denote values that are significantly different from one another within each variable type.

Content type and example				Frequency, n (%)		Omnibus chi-square (*df*)		*P* value
**Potentially misleading content (statements that could be readily interpreted in ways that result in misunderstanding or false conclusions)**				146 (100)		42.2 (6)		<.001
	Readiness of precision nutrition for application to individual diets	Diets can be truly personalized to one’s genes and this makes them more effective	56 (38)^‡^					
	Workings of nutrigenomics	Diet or food changes one’s genes	41 (28)^†,§^					
	Influence of genes on weight or size	Weight is mostly influenced by a single factor	23 (16)^‡^					
	Bad and good genes	One can have “bad genes”	15 (10)^‡,§^					
	A gene for...	Discussing “the obesity gene”	6 (4)^‡,§^					
	Specific business practice	Precision nutrition companies could sell DNA samples for nefarious purposes	4 (3)^†,‡,§^					
	Miscellaneous	—^a^	1 (1)^†^					
**Untrue content (statements that unambiguously state something that is untrue or unfounded)**				75 (100)		38.5 (6)		<.001
	Readiness of precision nutrition for application to individual diets	A personalized diet is the only diet that will be effective	11 (15)^§^					
	Workings of nutrigenomics	Specific dietary approaches can improve one’s DNA	48 (64)^‡^					
	Influence of genes on weight or size	Weight is not influenced by genes	5 (7)^†,§^					
	Bad and good genes	One’s genes will not allow them to gain weight	3 (4)^†,‡,§^					
	Specific business practice	Precision nutrition companies create diseases and spread them	3 (4)^†,‡,§^					
	Miscellaneous	—	5 (7)^†,‡^					

^a^Not available.

### Commercial Entities Referenced

Tweets in this data set referenced 54 unique commercial entities. Seven of these were referenced 5 or more times in this data set, and of those GenoPalate and Dynamic D Labs were referenced more than 20 times. Of the 54 commercial entities mentioned, 25 (46%) were mentioned in conjunction with potentially misleading information in at least 1 tweet 12 were mentioned in conjunction with untrue information in at least 1 tweet (Table S2 in Multimedia Appendix 1).

## Discussion

### Principal Findings

By evaluating social media discourse around precision nutrition on Twitter, we made several observations about the nature of the content available in a key environment through which individuals can learn about these concepts and products. The analysis sheds light on who is creating this information environment and the quality and reach of this information. Importantly, there is a relatively high rate of false information in relevant tweets, particularly in the context of nutrigenomic topics. This concerning finding is balanced by low rates of user engagement with tweets in our sample, those with and without false information, suggesting that misinformation is not being widely spread. Also promising is the finding of a substantial number of tweet authors with medical or scientific expertise who may be positioned to address misinformation when it arises or begins to spread.

### Tweet Authors

Overall, users who did not claim any scientific, medical, or wellness expertise were the most common tweet authors, which aligns with general usership statistics [51], although this varied among the tweet categories. Engagement rates with precision nutrition tweets were generally low across all categories, as 84.3% (370/439) had no replies, and the mean number of retweets was 1. This is consistent with generally low engagement tendencies across the wide swath of Twitter content [52]. A total of 14.9% (75/504) of tweets in this data set were noted to contain untrue information, which is also in line with studies that show a high prevalence of health misinformation on Twitter [40]. In this study, engagement with tweets containing untrue information did not differ from engagement with a general sample of tweets. This is encouraging given the concerns about the spread of health misinformation through social media [40]. In this case, although misinformation is certainly present, it did not spread beyond its initial introduction.

### Tweets About Products

Similar to previous investigations of scientific hype and premature translation [30], precision nutrition products were the most frequent focus of tweets. Although there was some informational content available in most product tweets, their ubiquity is likely related to the use of Twitter as a platform for informal advertising. This occurred despite the fact that data gathering procedures did not include “promoted tweets,” that is, paid advertising. In line with general advertising approaches, precision nutrition product tweets are more likely to link to a product directly and are framed most often in terms of advantages. Advantage framing is one component used in the identification of hype. Although mentions of disadvantages were fewer, they centered around interesting topics, such as privacy and misuse of genetic information. Anecdotally, disadvantage mentions did not tend to focus on the lack of scientific or clinical evidence to support current personalized nutrition claims. In fact, this topic rarely emerged at all in the sampled tweets. This suggests that knowledgeable audiences do not actively address the misleading tweets in this arena.

Product tweets were more likely to contain potentially misleading information. This tended to take the form of claims that one’s genes could be used to craft a truly personalized and effective diet. Tweets that focused on products were not, however, more likely to contain information that was untrue, suggesting that advertisements and discussions about these products did not tend to make patently false claims. Although tweets about products were not more likely to come from authors with a particular expertise profile, users without stated expertise were unlikely to link to products, suggesting a lack of organic discussion about precision nutrition products. Notably, engagement with product tweets was relatively low and did not differ from other categories. Several companies tweeted repeatedly and had very low engagement, suggesting spam-type advertising. In general, this pattern of results seems at odds with longstanding popular notions that products in this sector are making rampant false or unsubstantiated claims about benefits and that the public is actively seeking out these products in advance of their readiness [53].

### Tweets About Nutrigenomics Concepts

Tweets focusing on nutrigenomics concepts were the next most common in our sample. These tweets were more likely to be authored by wellness-focused users, as they often used nutrigenomic content to promote the intake of certain foods, nutrients, or supplements. These tweets were also more likely to provide an author’s professional credentials and link to an information source. In other words, these tweets tended to include the hallmarks of credibility. This approach is common among web-based health messages intended to be persuasive. The inclusion of credibility markers has been identified as a strategy for spreading health misinformation [54]. In this case, the largest proportion of untrue information was also found in nutrigenomics concept tweets, with a full 65% (48/75) of tweets containing false information falling into this category. Among all nutrigenomics tweets, 25% (32/128) were deemed to be false. This figure is somewhat lower than the limited evaluation of nutrition-focused misinformation on social media. A recent systematic review revealed that 62.5% of *studies* investigating nutritional information quality on social media classified information quality as “poor” [55]. A study of nutrition tweets posted in Arabic rated 36% of the sample tweets as “false” [56].

### Tweets About Genetics and Body Weight or Size

The other very prevalent category is the discussion of the role of genetics in body weight or size. This tweet type mostly authored by users who did not claim science, medical, or wellness expertise. Although this is more tangential to the precision nutrition landscape, searches used to seek information about nutrigenetics or omics will include information on this topic. It has also been suggested that discourse on the role of genetics and lifestyle in weight is an important precursor to understanding consumer responses to nutrigenetics [57]. More broadly, the role of genes in weight is a popular topic of public discussion, and weight loss is a major topic of conversation on Twitter [58]. This topic is also commonly identified in other social media outlets and investigations of lay audiences [59,60]. Tweets about genetic influence on body weight or size tended to be more casual and discussion-based and relied on lay notions of genetic influences on weight. The prevalence of this topic in the current analysis suggests that social media users think about and discuss the role of genes in weight and related processes. In contrast, the role of genes in eating behavior, was rarely discussed. This is likely because among lay audiences, the role of genetics in influencing eating behaviors tends to be less salient than the role of genes in influencing weight [61]. Twitter also does not appear to be a major source of information or discussion about nutrigenetic concepts outside product-based tweets. This is likely because nutrigenetics (aside from product-based content) is fairly specific and relies on relatively complex concepts.

### Untrue and Potentially Misleading Content

The number of tweets identified as containing content that was untrue or potentially misleading appears to be quite high, although these rates are difficult to contextualize. This topic of discussion does indeed appear to be fertile ground, given the complexity of science and the commercial interests involved. Although misinformation regarding precision nutrition is therefore prevalent on Twitter, it is mostly aligned with wellness and nutrigenomics. Many of these tweets focused on the idea that diet or nutritional supplements influence genes. The presence of false nutritional information and unsupported claims on social media has been documented in many other domains including cancer, diabetes, and pregnancy, and has been linked with adverse health outcomes among consumers [62-64]. The potential downstream influences of the misinformation documented here on consumer health are unclear but should be considered if and when discourse in this area grows.

We additionally flagged potentially misleading information in the current analysis to highlight areas that might lead to confusion regarding the nature of nutrigenetics and nutrigenomics; however, the nature of Twitter (eg, character limits) often necessitates imprecise language. As such, instances of potentially misleading content are expected on this platform and may not be a cause for serious concern. The most prevalent examples of potentially misleading content are those suggesting that diet influences “DNA.” Although this could be considered a shorthand for describing epigenetic processes (ie, elements of diet influence gene expression), it could alternatively be interpreted as diet altering one’s actual DNA. The presence of untrue information is more concerning as it involves incorrect “facts.” The potential harm of such misinformation likely relate to the particulars of each tweet and cannot be generalized across the data set given the wide range of topics involved. That said, tweets with incorrect information did not tend to engender any additional engagement and thus were unlikely to have a broad influence. This analysis highlights topics that may require informed voices to correct misconceptions and provide reputable and correct information to enhance public understanding of genetics, genomics, and nutrition. Authors with scientific or medical expertise tended to engender more engagement in the current analysis, with a higher number of retweets and likes per tweet. Although there were no significant differences in the number of followers between categories, this metric was highly skewed, with some authors in this analysis exceeding a million followers.

### Limitations

This study had several limitations. First, the sample was limited to a particular time frame to obtain a manageable sample of tweets for coding. We judged the timeframe we chose to be unremarkable in terms of any events or news items related to the topics at hand. The popularity of topics and nature of discourse tend to change over time. Although we are unaware of any events in precision medicine that would likely cause a great shift in this discourse, the nature of Twitter as a platform has changed over time given changes in ownership and business models, which may affect these discussions in unknown ways. The sampling period also occurred during the COVID-19 pandemic, which may have shifted the conversation away from non-COVID topics such as nutrigenetics. In general, the pandemic increased participation on Twitter and other social media platforms [63], and while much of the increased traffic pertained to COVID-19, discourse around other health topics persisted. Data from an analysis of Twitter discourse around autism spectrum disorder, for example, suggest that non-COVID autism topic structures remained similar to prepandemic structures [65]. Second, engagement with precision nutrition topics was found to be fairly low in this analysis, which is an important finding as well as a limitation of this study. We embarked upon this study because there was no existing literature in this area, and thus, it was not characterized enough to have a sense of the frequency and reach of these topics on social media before we began the analysis. The limited number of tweets identified may be due, in part, to the search terms, which were relatively technical and scientific. This approach may have missed more generally worded tweets that addressed these concepts. Technical terms do tend to work their way into common language as technologies become more entrenched, and as such, we believe that the current findings are a useful benchmark to which future work can compare.

Third, the analysis included over 500 tweets, which is on the smaller side for social media analysis, but is typical when deep hand-coding-based content analysis is performed. For categories with fewer exemplars, the distribution of characteristics may be less representative of the larger tweet population. Emerging artificial intelligence analysis approaches may be capable of deep characterization of tweets in a larger sample. Fourth, we limited the analysis to include content related to precision nutrition in the context of genetics and genomics. Other approaches involving the use of metabolites and biomarkers are also increasing in popularity, and the discourse around these approaches may differ. Although this was beyond the scope of the current analysis, future work should explore these discussions. Finally, although we fact-checked tweet content, we took other information types largely as written and did not question assertions made by tweet authors (although we did actively account for sarcasm, humor, etc). As such, some of the biographical and other information provided by users may not be factual; anyone can claim expertise on Twitter. In the current analysis, we were more interested in noting markers of authority and expertise than in identifying tweet authors’ actual identities.

### Conclusions

Twitter does not appear to be a major source of discussion regarding precision nutrition during this time, given low engagement metrics in the sample analyzed here. It is unclear whether there are other venues for these conversations or whether they are generally uncommon. To the extent that these discussions occur, we were able to distinguish several patterns that characterize the conversation and identify patterns related to source expertise, positive or negative framing, commercial activities, and information quality.

Overall, the tweets that were analyzed had low engagement across the board; however, there was evidence of engagement among a contingent of users with scientific and medical expertise who were active in discussing nutrigenomics concepts and products. This user population may be encouraged to share credible expert advice on precision nutrition and tackle false information. Indeed, there is evidence that reputable health voices can successfully counter misinformation on social media [45].

For consumers interested in finding credible information about products related to health and weight loss, investigation of the broader topic of precision nutrition is needed. The landscape of precision nutrition is predicted to change substantially in the coming years owing to advances in science and medicine. As this occurs, it is crucial to continue to evaluate the information environment in which patients, consumers, and other stakeholders seek and are exposed to nutrigenetics and nutrigenomics-related concepts. By working to inject credible, evidence-based content into this environment, we may also help these stakeholders avoid misunderstanding and the use of products that are not yet ready to provide actionable dietary advice.

## Appendix

Multimedia Appendix 1 Study codebook and table of commercial entities.

## Data Availability

The data sets generated and analyzed during this study are not publicly available because of the identifiable nature of the primary tweet and author content but are available from the corresponding author upon reasonable request.
