# Supplementary material for: Characterizing Precision Nutrition Discourse on Twitter: Quantitative Content Analysis
Source: J Med Internet Res. 2023 Oct 12;25:e43701. doi: 10.2196/43701 (PMC10603558; doi:10.2196/43701)
Supplement: Multimedia Appendix 1 [file jmir_v25i1e43701_app1.docx]

**Supplemental Table 1 – Codebook**

| General:   - Quoted tweets are to be considered part of the tweet unit, however, quoted materials should be considered in light of what the poster is saying - If tweet is part of a thread, code each tweet that occurs in data set individually. Use rest of thread for context - Code only English tweets; one short phrase in another language is ok - Hashtags and tags/mentions can be treated as content or context, whichever makes sense based on their position in the larger tweet. | | |
| --- | --- | --- |
| **CATEGORY** | **DEFINITION** | **INSTRUCTION** |
| **TWEET CATEGORY** | Categories mutually exclusive - pick the best category. If truly more than one category, categories are ordered by importance, so choose category higher on list.  In order to categorize:   - Must refer to human genetics, not agriculture or animal genetics - Tweets that refer to a gene relating to a disease ineligible unless disease is obesity or eating disorders (see below) - Use only what is seen in the initial tweet to assign category. - Interpret jokes/sarcasm the best you can to be consistent with the meaning. - Don’t include genetics used as idioms/turns of phrase (e.g., she has cooking in her DNA). - Tweets can be included in a category when the category is negated (e.g., genes don’t influence body weight) - Coder bio can be used as context, e.g., if it’s a genetic testing company tweet can be viewed in light of this | |
| 1. Nutrigenetics/omics Product | Reference to a specific product(s) or commentary (positive or negative) on a specific product though product does not need to be named   - Mention of genetic test + a word like: lifestyle, wellness synonym, prevention, optimize should be coded - Do not include simply the concept of personalizing a diet - Mention of a specific test can be in the hashtags/tags - Nutrigenomics products should be marketed as something that will change your DNA (e.g., supplements) to be included - If a company is tweeting it’s not automatically in this category, but it is likely | Category = **1** |
| 1. Nutrigenetics concepts | DNA influences response to nutrients in the diet and the body’s response   - Can also relate to genetic influence on what the body does in absence of food | Category = **2** |
| 1. Nutrigenomics concepts | What someone eats influences gene expression or DNA characteristics;   - Also count changes in offspring DNA through epigenetics | Category = **3** |
| 1. Genes influence eating behavior/food preference | Genes influence food choices/preferences/experience of food, when/why/how/where/who of eating   - Including eating disorders | Category = **4** |
| 1. Genes influencing body weight/size | Direct genes to weight relationship, does not include the interplay between genes and food   - Physique; muscle size also count as body ‘size’ - Also select this category if tweet states genes do not influence body size/weight | Category = **5** |
| 1. Other | Must still pertain to relationship between food and genes.   - For example – use other when the word “nutrigenetics” or “nutrigenomics” is used, but not in an otherwise codable context | Category = **6** |
|  | | |
| **AUTHOR** | **Who is tweeting?**   - Mutually exclusive, choose the best answer - If unclear can click on twitter-provided links but do not go beyond provided links |  |
| 1. Business | Tweet originates from a for profit business   - mark all businesses even if not engaged in DNA testing | Category = **1** |
| 1. Academic/non-profit/ educational institution | Tweet originates from an account representing an academic institution, educational institution, non-profit, health industry group, or similar | Category = **2** |
| 1. Individual | Tweet originates from an individual who is not associated with a company.   - If someone uses their name assume it is an individual - Count as individual if tweeting under own name even if they own a company or are affiliated with a larger entity | Category = **3** |
| 1. Media | Tweet originates from an account representing a media source, include blogs, content aggregators, journals, etc. | Category = **4** |
| 1. Other |  | Category = **5** |
|  | | |
| **AUTHOR CREDIBILITY** | - Take stated information at face value |  |
| Credentials that are relevant | Author holds academic/medical credentials and puts this information in bio/name. not a general title that usually has credentials (research scientist) unless it is specific physician type (e.g., psychiatrist)  Ex:   - M.D. - PhD - RD - RN - “Dr” in their bio name | 1=yes  0=no  NA = for an institution |
| Institutional affiliation | Author notes an affiliation with an academic, government, legitimate media, scientific journal, scientific body, or other non-corporate/neutral affiliation   - Regardless of nature of affiliation (include affiliation for students) | 1=yes  0=no |
| Implied/Apparent Scientific or Medical Expertise | Author/entity has apparent or implied expertise related to the information in the tweet and that expertise is scientific or medical in nature (as opposed to wellness).   - Institutional label implies expertise (e.g., health policy center; County Hospital) - Include reporters with specialty in medicine/science/health etc. - Company who offers an expert service related to science/medicine is claiming expertise in that service - Related words on their own (science, nutrition) aren’t enough without context suggesting there is expertise there | 1=yes  0=no |
| Implied/Apparent Wellness Expertise | Author/entity has apparent or implied expertise or holds a specific certification related to wellness. Could include: wellness coach, health coach, self-help, fitness specialist, fitness trainer, etc.   - Institutional label implies expertise (e.g., the wellness center; Gold’s Gym) - Applies to companies who work in this space – this implies expertise in that service (e.g., fitness) - Wellness words are ok – for example: health, chemtrails, keto - Include weight loss companies here unless clearly medical - Personal weight loss not sufficient to indicate expertise | 1=yes  0=no |
|  |  |  |
| **INFO CREDIBILITY** |  |  |
| Credibility References | Tweet references credible source of information/support – e.g. journal article, reputable organization, mainstream media, or experts/professionals as support for tweet   - Can be in the body of the tweet or can be in a link posted as part of the tweet - Count as credible if source has name recognition (use Wikipedia for foreign news sources to assess stats as major/mainstream news) - Count as credible if a link is written by authors with credentials, cites an academic study | 1=yes  0=no |
|  |  |  |
| **ENGAGEMENT** | - Tweet must be at least 2 weeks old for engagement content to be evaluated - Collect data directly from tweet; if it is deleted use N/A |  |
| Retweets | - Count both retweet and quote retweet together | Number |
| Likes |  | Number |
| Replies | Response to tweet   - Count self-replies - Count deleted replies | 0=0  1= <10  2= ≥10 |
|  | | |
| **CONTENT** |  |  |
| Stated advantages of nutrigenetics/omics | Tweet states something specific and positive about a product or the application of information/knowledge about the relationship between genes and food  Look for stated or heavily implied advantage for example:   - Stating that nutrigenetics/omics are legitimate for promoting health - Supporting/marketing use of use of genetics/food applications - Most ads will count here unless they aren’t making any claims - The advantage does not need to be specific to nutrigenetics, for example, reducing self-blame or making people happier would count | 1=yes  0=no |
| Stated problems | Tweet states something specific and detrimental/negative about a product or the application of information/knowledge about the relationship between genes and food  Look for stated or heavily implied disadvantages for example:   - Stating that nutrigenetics/omics are not legitimate for promoting health, or that the science is not ready for application or that it can’t be used - Suggesting people should not use genetics/food applications - The disadvantage does not need to be specific to nutrigenetics, for example, violating privacy would count - Questioning or disagreeing with gene-food counts (e.g., genetics does not influence weight) | 1=yes  0=no |
|  | | |
| **LINKS** |  |  |
| Link to product or service | Tweet links to a genetic, dietary, lifestyle, wellness, or related product, service, etc.   - Link must be direct to the product/service site - Web links only, not tags - Count links that are expired (e.g., ebay listings) as if they are there - Count indirect links to product if there is a link to ad or infomercial-type content that then links to product for purchase, or if links to a coupon that then can be used for purchase | 1=yes  0=no |
| Links to an informational resource | Tweet links to an information resource related to the genetic/diet element of the post in some way   - Need not be legitimate media - Include visible links in the quoted tweets - Only review the content that was on the page that the tweet linked to; don’t go further into the website - If a product – looking for factual information about the product/health conditions etc. rather than just links to products and sales-y language | 1=yes  0=no |
| Companies or products mentioned | Collect names of nutrigenetics/omics products/companies   - Include companies in linked information source | Record name of company and/or product |
|  |  |  |
| **MISLEADING OR INCORRECT** | - Mutually exclusive – if tweet contains any incorrect information code as incorrect as opposed to potentially misleading even if it contains both - If there is any doubt employ fact checking |  |
| Potentially misleading | Tweet contains information that is not technically correct and may be misinterpreted to cause or reinforce misinterpretations related to nutrigenetics/omics, related companies, etc. | 1= yes  0 = no |
| Potentially misleading category | - Mutually exclusive, choose the best one   1. Related to diet personalization - there is currently the ability to personalize diet using genetics in a way that works or can be optimized for individuals. Also taking population-level findings and applying to an individual for prediction  2. Related to foods altering DNA without specifying epigenetic mechanisms  3. Foods affect specific biological processes (in ways that aren’t scientifically supported)  4. Effects of GMOs on biology/DNA (in ways that aren’t scientifically supported)  5. A single factor (e.g., genes) are much more important for weight than others  6. The notion of “good” or “bad” genes  7. The notion there is “a gene for” a disease (e.g., the obesity gene)  8.Reference to business practices of a related company (that aren’t supported by other sources)  9. Other | Category number |
| Untrue | Tweet contains information that is demonstratably false | 1= yes  0 = no |
| Untrue category | - Mutually exclusive, choose the best one   1. Related to diet personalization - personalized nutrition is the best or only diet that will work; DNA analysis will provide list of specific foods to eat or not eat (unless specific food supported by evidence)  2. Related to foods altering DNA in specific ways including making improvements or causing damage  3. Foods affect specific biological processes (in ways that aren’t scientifically supported)  4. Specific effects of GMOs on biology/DNA (in ways that aren’t scientifically supported)  5. Stating weight is not influenced by genetics or is only influenced by genetics  6. -  7. -  8.Reference to business practices of a related company (that aren’t supported by other sources)  9. Other | Category number |

**Supplemental Table 2 – Commercial entities referenced in tweets**

| **Commercial entity** | **Number of Tweets (% of total)** | **N with potentially misleading information** | **N with untrue information** |
| --- | --- | --- | --- |
| 23& Me | 7 (1.4%) | 4 |  |
| Ancestry D | 2 (0.4%) | 2 |  |
| ARCpoint Labs | 12 (2.4%) | 11 |  |
| Athletigen | 1 (0.2%) | 1 |  |
| BaleDoneen Method | 1 (0.2%) | 1 |  |
| Charge Products | 1 (0.2%) |  | 1 |
| Culinary Genes | 1 (0.2%) | 1 |  |
| CRIgenetics | 1 (0.2%) |  |  |
| GenoPalate | 21 (4.2%) | 4 | 5 |
| Nutrigenomix | 3 (0.6%) | 1 |  |
| D Nudge | 1 (0.2%) |  |  |
| D Fit | 2 (0.4%) |  |  |
| Nestle Wellness | 1 (0.2) |  |  |
| Genesis Healthcare | 1 (0.2) |  |  |
| d frikasa | 1 (0.2) | 1 |  |
| d Power | 1 (0.2) | 1 |  |
| D Gundry’s Diet Evolution | 1 (0.2) | 1 |  |
| Dynamic D Labs | 21 (4.2%) | 1 |  |
| Energybits | 1 (0.2%) |  | 1 |
| Foodmonster/One Green Planet | 7 (1.4%) |  | 7 |
| Gene Eating | 1 (0.2%) |  |  |
| Genefitletics | 2 (0.4%) |  |  |
| Genes2Me | 1 (0.2%) | 1 |  |
| Genomic Kitchen | 9 (1.8%) | 4 | 1 |
| Gousto D Dishes | 1 (0.2%) |  |  |
| GX Sciences | 1 (0.2%) |  |  |
| AGX | 1 (0.2%) |  |  |
| Pathway Genomics | 1 (0.2) |  |  |
| Fitgenes | 1 (0.2%) |  |  |
| LIFEdata | 1 (0.2%) |  |  |
| GxSlim | 1 (0.2%) |  | 1 |
| HomeD | 4 (0.8%) |  |  |
| iweightlossnow | 1 (0.2%) | 1 |  |
| K&H Personalized Medicine | 3 (0.6%) |  |  |
| Levels | 1 (0.2%) |  |  |
| Lifecode Gx | 2 (0.4%) | 1 |  |
| LifeD | 1 (0.2%) |  | 1 |
| MCBodyClinic | 1 (0.2%) | 1 |  |
| Medcomp Sciences | 1 (0.2%) | 1 |  |
| Muhdo | 2 (0.4%) |  | 2 |
| NGX Nutrition | 1 (0.2%) | 1 |  |
| PinkFiit | 3 (0.6%) | 2 | 1 |
| Planet Naturopath | 5 (1.0%) | 2 | 2 |
| Qi | 1 (0.2%) |  |  |
| Ripesey | 4 (0.8%) | 3 |  |
| Sano Genetics | 1 (0.2%) | 1 |  |
| Sunwarrior | 1 (0.2%) | 1 |  |
| Suprastatum | 1 (0.2%) |  |  |
| Sushi Singularity | 1 (0.2%) |  |  |
| Suspire Labs | 1 (0.2%) |  | 1 |
| Thrivous | 1 (0.2%) | 1 |  |
| Uforia | 1 (0.2%) |  | 1 |
| Xcode Life | 2 (0.4%) |  |  |
| Your Gene Team | 1 (0.2%) |  |  |
